# A Highly Efficient Workflow for Detecting and Identifying Sequence Variants in Therapeutic Proteins with a High Resolution LC-MS/MS Method

**DOI:** 10.3390/molecules28083392

**Published:** 2023-04-12

**Authors:** Lance Cadang, Chi Yan Janet Tam, Benjamin Nathan Moore, Juergen Fichtl, Feng Yang

**Affiliations:** 1 Pharma Technical Development, Genentech, South San Francisco, CA 94080, USA; cadang.lance@gene.com (L.C.);; 2 Pharma Technical Development, Roche Diagnostics GmbH, 82377 Penzberg, Germany

**Keywords:** sequence variants, sequence variant analysis, mass spectrometry, high-resolution, LC-MS/MS, protein therapeutics, minor variants, peptide mapping

## Abstract

Large molecule protein therapeutics have steadily grown and now represent a significant portion of the overall pharmaceutical market. These complex therapies are commonly manufactured using cell culture technology. Sequence variants (SVs) are undesired minor variants that may arise from the cell culture biomanufacturing process that can potentially affect the safety and efficacy of a protein therapeutic. SVs have unintended amino acid substitutions and can come from genetic mutations or translation errors. These SVs can either be detected using genetic screening methods or by mass spectrometry (MS). Recent advances in Next-generation Sequencing (NGS) technology have made genetic testing cheaper, faster, and more convenient compared to time-consuming low-resolution tandem MS and Mascot Error Tolerant Search (ETS)-based workflows which often require ~6 to 8 weeks data turnaround time. However, NGS still cannot detect non-genetic derived SVs while MS analysis can do both. Here, we report a highly efficient Sequence Variant Analysis (SVA) workflow using high-resolution MS and tandem mass spectrometry combined with improved software to greatly reduce the time and resource cost associated with MS SVA workflows. Method development was performed to optimize the high-resolution tandem MS and software score cutoff for both SV identification and quantitation. We discovered that a feature of the Fusion Lumos caused significant relative under-quantitation of low-level peptides and turned it off. A comparison of common Orbitrap platforms showed that similar quantitation values were obtained on a spiked-in sample. With this new workflow, the amount of false positive SVs was decreased by up to 93%, and SVA turnaround time by LC-MS/MS was shortened to 2 weeks, comparable to NGS analysis speed and making LC-MS/MS the top choice for SVA workflow.

## 1. Introduction

Protein biotherapeutics such as monoclonal antibodies (mAb), enzymes, and other recombinant proteins are typically manufactured using mammalian (HEK 293, CHO) [1,2,3,4] or bacterial (*E. coli*) [5,6,7] cell culture. While these expression systems are great at producing these proteins at an industrial scale [8], they make a heterogeneous protein that includes not only the desired protein but also minor and potentially undesired variants [9,10]. One such minor variant is unintended amino acid substitutions, more commonly known as sequence variants (SVs) [11]. The misincorporation of amino acids can result from either mutation in the product DNA template within the host cell or errors in the cells’ protein translation machinery [12]. Due to their potential impact on patient safety and product efficacy, SVs need to be fully characterized, measured, and controlled [13,14].

DNA sequencing techniques such as Sanger sequencing or Next-generation Sequencing (NGS) can detect and quantify genetically induced sequence variants in production cell lines with reported sensitivities of ≥5% and ≥0.5%, respectively [15]. NGS also has the advantage of only requiring a small amount of hands-on laboratory time and automated data analysis [15]; however, it only detects mutations in the DNA but not at the protein level. In contrast, liquid chromatography-tandem mass spectrometry (LC-MS/MS) can detect and quantify both genetically derived and translational error-derived SVs since it characterizes SVs at the protein level [16]. Yang et al. previously reported the LC-MS/MS method to detect and quantify sequence variants [17]. This approach combined tryptic and chymotryptic mapping using low-resolution ESI-ion trap MS/MS combined with Mascot Error Tolerant Search (ETS) [17]. In a given sequence variant analysis (SVA) experiment, this approach can generate up to 1000 potential SV hits. Applying a simple filter for common false positives such as amino acid additions, improperly cleaved peptides, and relative ratios below a certain threshold can eliminate 40–60% of these potential SV hits but still leaves the experienced MS data analyst hundreds of SV hits for manual inspection [18]. This tedious analysis can take 6–8 weeks from sample submission to data release.

Most of the false positive SV hits have come from misidentifications caused by the low-resolution, high-mass-error tandem mass spectra (MS/MS) generated by the linear ion trap mass analyzer. Lowering the mass error tolerance during MS/MS ETS removes most of these false positives. With the upgrade of the LTQ linear ion trap to the Orbitrap Elite hybrid instrument, the resolution of the MS1 precursor scan can be dramatically increased. While the Orbitrap mass analyzer boasts superior mass resolution compared to that of the linear ion trap, it is also significantly slower [19], which translates to longer duty cycle times, the amount of time required to finish one cycle of MS1 and MS2 scans [20], nd a reduced number of MS1 scans that can be obtained for each peak. A good data-dependent acquisition (DDA) experiment contains a sufficient number of MS1 scans per peak while being able to obtain MS2 on all lower-level components that are at or above the defined sensitivity requirement [21]. In our experience, a minimum number of eight to ten MS1 scans per peak is required to ensure enough data points to perform quantitation by peptide map. Switching both MS1 and MS2 to the Orbitrap will ultimately lead to poorer sensitivity and badly extracted ion chromatogram (XIC) peaks due to the significantly decreased number of precursor scans. For this instrument, the ideal DDA peptide mapping method was to have the MS1 performed in the Orbitrap while the MS2 occurred in the linear ion trap [22].

Over the past few years, there have been significant improvements in mass spectrometry hardware. The scan rates of Orbitrap mass analyzers equipped within the Q-Exactive, Fusion, Fusion Lumos, and Exploris models have increased to a point where it is now possible to perform both MS1 and MS2 in the Orbitrap [23,24,25]. These new models can do a self-internal calibration (Easy- IC) if they are equipped with an ETD source, which uses the ETD reagent fluoranthene as the lock mass in each scan [24]. With internal calibration, these instruments can achieve mass errors <1 ppm in every scan consistently and without the need for an additive compound in the mobile phase [26]. Because the newer platforms have vastly different equipment for ion optics, focusing, and transfer, a full MS method development including cross-comparison to the previous method is required.

An efficient way to evaluate key MS instrument parameters and possible parameter interactions in a statistically meaningful way is through a Design of Experiments (DOE) approach [27]. A statistical DOE uses replication, blocking, randomization, and orthogonality to probe the interaction of variables and uses statistics as an objective way to draw conclusions while taking into account errors, noise, and possibly other unknown variables [28]. This approach has been successfully used in LC-MS applications before being included in the optimization of the ESI source interface [29], instrument run times [30], and development of generic methods to optimize both LC and MS parameters simultaneously [31]. Once the MS parameters are established, an evaluation will be made based on the sensitivity, the number of SV hits generated, and the relative quantitation of previously identified SVs from previously analyzed samples. In this study, we aimed to develop a method that can achieve a similar LOD of at least 0.2% while dramatically decreasing the amount of SV candidates for manual verification. During the development work, we also discovered a new setting within the Fusion Lumos instrument that was causing some issues with the relative quantitation of SVs and PTMs, highlighting the importance of doing thorough development and comparison studies before switching to the new method.

Another way to improve SVA workflow is through data analysis. Our previous method used Mascot-ETS and Protein Metrics (PMI) Integrated Software for Sequence Analysis (ISSA) software, which is the precursor to PMI Byologic. This method relied on manual input of processing parameters each time an SVA experiment was performed. It required .raw file conversions to .mgf using ProteoWizard, an upload of each generated file for a regular Mascot search, and then a reprocessing of the search file with a Mascot ETS. After this, we copied and pasted the generated links from the mascot server into the ISSA setup screen, which also did not have pre-defined workflows. Overall, this process to set up the data analysis file used to take an entire workday to complete. Since all of the parameters were manually entered, a reviewer also needs to take time and pay close attention to the parameters used to make sure that they were correct. If there was a mistake in the parameter input, then the process will have to start from the beginning. Protein Metrics Byos software provides an integrated solution to peptide map data analysis. This has the advantage of using pre-defined workflows for PTM/SV data analysis that already contains the parameters used for the XIC and MS/MS database search. This software also has an interactive user interface that enables an analyst to inspect key attributes of a potential SV hit such as MS2 quality, precursor mass error, monoisotopic mass arrangement, and retention time shift in one screen. Relative quantitation of identified true positive SVs are performed within the same program and can be recorded via automatically generated reports [32].

In this work, we present an improved SVA method by high-resolution LC-MS/MS combined with Protein Metrics (PMI) Byos’ integrated SVA workflow. We first used the Design of Experiments (DOE) approach to efficiently determine the optimized instrument parameters and probe possible multi-way parameter interactions and the use of the Easy-IC internal calibration feature of the instrument. We also discovered a particular parameter within the Fusion Lumos MS that caused major differences in quantitation and further acquired data from other common high-resolution Orbitrap instruments to compare the relative quantitation values. In addition, we established a score cutoff of PMI’s Byonic MS/MS search algorithms for SVA. Our new SVA workflow has led to the greatly increased SVA speed comparable to NGS and can detect SVs from either genetic mutation or translation errors, making LC-MS/MS a top choice for SVA.

## 2. Results and Discussion

### 2.1. Parameter Screening DOE Study

**Figure 1:**
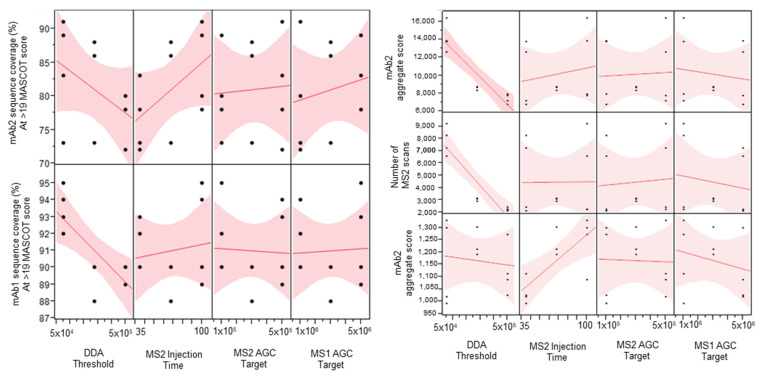
Scatterplot matrix showing the main effects of each factor on sequence coverage, aggregate score, and number of MS/MS scans.

The four-factor half-factorial screening design explored the main effects of each of the tested factors while allowing for analysis of possible two-way interactions. Data-dependent acquisition (DDA) intensity threshold is the minimum intensity at which an ion can be selected for tandem mass spectrometry. MS/MS injection time is the maximum amount of time allowed for the selection and injection of the precursor ion into the tandem mass spectrometry scan. The MS/MS AGC target is the maximum number of ions allowed to enter the trap in a given scan. MS/MS injection time and MS/MS AGC targets are related parameters. The MS1 AGC target is the maximum number of ions allowed in the Orbitrap during the MS1 full scan. The main effects of each of the tested factors are shown in the scatterplot matrix (Figure 1).

DDA intensity threshold was shown as the most important factor among the four tested factors (Figure 1). Increasing this parameter had an adverse effect on the sequence coverage of both the low-abundance (mAb1) and high-abundance (mAb2) proteins. The high DDA intensity threshold also significantly decreased the total number of MS/MS scans obtained, which then decreased the number of PSMs and caused the abundant protein aggregate score, the sum of the scores of the PSMs found by Mascot, to decrease. In this case, we use the aggregate score as a rough measurement of overall spectral quality. The MS/MS injection time seemed to have a positive effect on the lower abundance protein aggregate score while it did not affect that of the higher abundance protein. This suggests that using higher injection times might help produce better quality MS/MS spectra for low abundance ions, which is important to determine the exact amino acid position of sequence variants. An additional single-factor screen was performed to evaluate the effect of even higher MS/MS injection times on sequence coverage and aggregate score. Higher than 150 ms injection times started to show diminishing returns on the sequence coverage and aggregate scores (Table 1). The MS1 and MS/MS AGC targets did not seem to have much effect on the response factors tested. A Pareto analysis of the results did not show any significant two-way interactions (Supplementary Figure S1).

Based on the results of the screening experiment, the following conditions were selected for the high-resolution SVA method: low DDA intensity threshold, high MS/MS injection time, and low instrument AGC targets for both MS1 and MS/MS. For the DDA intensity threshold, 5 × 10^4^ was selected since this is the minimum manufacturer-suggested threshold for orbitrap MS/MS analysis. The injection time of 120 ms was selected since this was the maximum injection time where there could still be a minimum of eight MS1 scans across each chromatographic peak. It is important to have at least eight MS1 scans in each peak for XIC in order to get an acceptable peak shape and a more robust quantitation.

### 2.2. Fixing the Under-Quantitation in the Fusion Lumos

**Figure 2:**
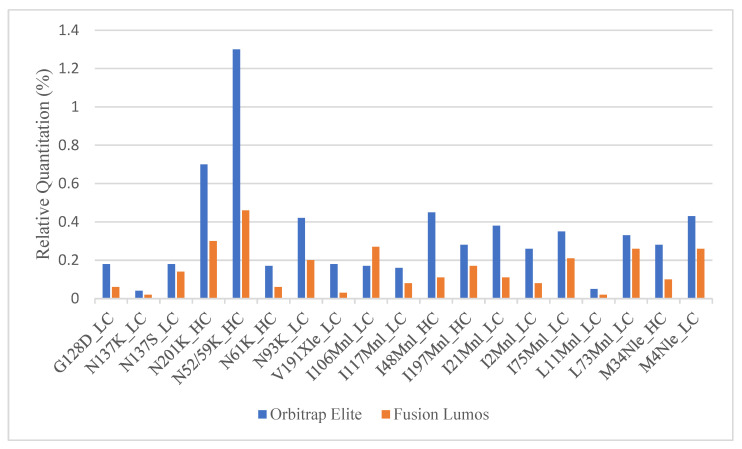
Comparison of the relative quantitation of true positive sequence variants obtained in Orbitrap Elite vs. Fusion Lumos.

Once the MS parameters on the Fusion Lumos were determined, a side-by-side comparison between the current and the new method was performed. A full SVA analysis of a fab fragment was run in both the Fusion Lumos and the Orbitrap Elite. While the new method was able to detect all true positive sequence variants with much fewer potential SV hits (471 new vs. 1441 current), the relative quantitation calculated on the Lumos was roughly two- to three-fold lower than that obtained in Orbitrap Elite (Figure 2). Multiple parameters including H-ESI source settings, MS1 resolution, MS1 injection time, MS1 AGC target, MS2 resolution, MS2 AGC target, protein injection load, column length, and instrument cleanliness were tested in single-factor experiments to see if any of these resulted in higher relative quantitation of the SVs. None of the parameters enumerated were able to meaningfully change the relative quantitation of low-level peptides.

An MS1-only experiment and a Top 5 DDA experiment were performed using 0.5% mAb1 spiked in mAb2. The results showed that the MS1-only experiment yielded a ~0.5% relative quantitation of the lower-level peptide, while the Top 5 DDA experiment yielded ~0.3%, which is similar to the observed difference between the Orbitrap Elite and the Fusion Lumos SVA results. In order to further understand this phenomenon, a concurrent method where an MS/MS experiment is stacked with an MS1-only experiment was tested with the SST sample. The results from the concurrent experiment confirmed the phenomenon observed in the separated MS1-only and DDA experiments. Furthermore, it suggested that only the high abundance peptide was affected and caused the quantitation differences. In this experiment, we observed that while the peak intensities of the peptides coming from the less-abundant mAb1 were similar between the MS1-only and the DDA experiments, the peak intensities of the peptides coming from the more abundant mAb2 increased by approximately twofold in the DDA experiment.

**Figure 3:**
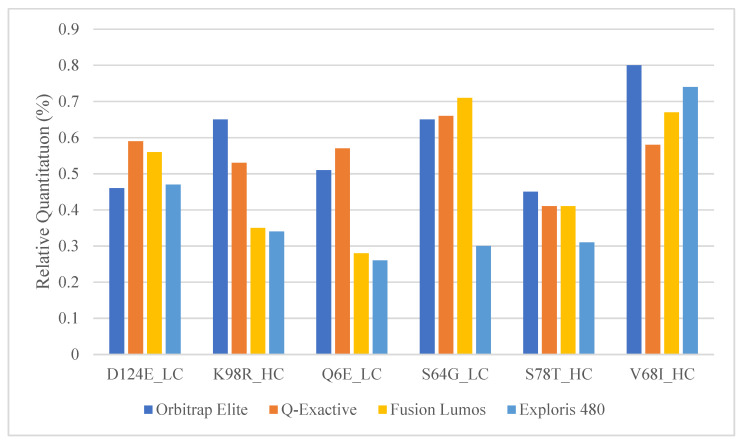
Relative quantitation of the mAb1 and mAb2 peptide pairs in commonly used Orbitrap platforms.

The research and development team of the instrument vendor was consulted to further understand the issue and determine the root cause of the abundant peptide signal bias. It was eventually determined that the main culprit causing the signal bias is a setting called Isotope Interaction Threshold (IIT). IIT is a feature of the Fusion Lumos designed to prevent significant space–charge interactions in the Orbitrap mass analyzer. IIT dramatically lowers actual MS1 injection fill time if a mass error above a certain value is detected due to significant space–charge interactions. MS assays with two or three dominant species per scan such as mAb peptide maps are prone to such space–charge interactions. A low IIT setting leads to an extremely low actual injection time, which results in a disproportionate increase in abundant ion signal. This is because the signal intensity is equal to the number of detected ions in the Orbitrap divided by the actual injection time, and abundant ions fill the trap quicker than low-level ions. The default IIT setting for the Fusion Lumos was 0.75 ppm, which is extremely low. In order to fix the quantitation issue, the IIT setting was increased to 100 ppm, which essentially turned the feature off. This resolved the under-quantitation issue observed in the Fusion Lumos (Figure 3). Furthermore, the vendor recommended that special attention be given to AGC targets and protein loads in order to make sure that MS data was not compromised by space–charge interactions. We checked for potentially increased mass errors that might happen due to space–charge interactions, but none were observed. For the subsequent testing, we lowered the AGC target from 1 × 10^6^ to 2 × 10^5^ per the vendor’s recommendation.

To test if this new method was sensitive enough to detect sequence variants at an LOD of 0.2%, a sample with 0.1% mAb1 spiked into mAb2 was analyzed in the Fusion Lumos. The 0.1% spike sample showed a similar sequence coverage (at around ~92%) of mAb 1 compared to the 0.5% spike sample. Furthermore, the obtained relative quantitation values of the mAb1 and mAb2 peptide pairs in this 0.1% spike sample were close to the expected value of 0.1% (Supplemental Figure S2). This demonstrated sensitivity is comparable to previously reported SVA methodologies [15,17,33].

### 2.3. Method Robustness DOE

A four-parameter, half-factorial screening DOE study was performed to check method robustness with respect to MS1 and MS/MS Orbitrap resolution, MS/MS injection time, and fragmentation type. Given the scan speed and transient times of the MS instrument, we have chosen the maximum resolution and injection time that will provide sufficient sensitivity. The parameters were 120 K FWHM for MS1 resolution, 60 K FWHM for MS/MS resolution, and 120 ms for MS/MS injection time. These target conditions will be the midpoint of the robustness experiment. A fragmentation type parameter was also added to provide additional flexibility in case a switch to Higher-energy C-trap dissociation (HCD) was warranted.

**Figure 4:**
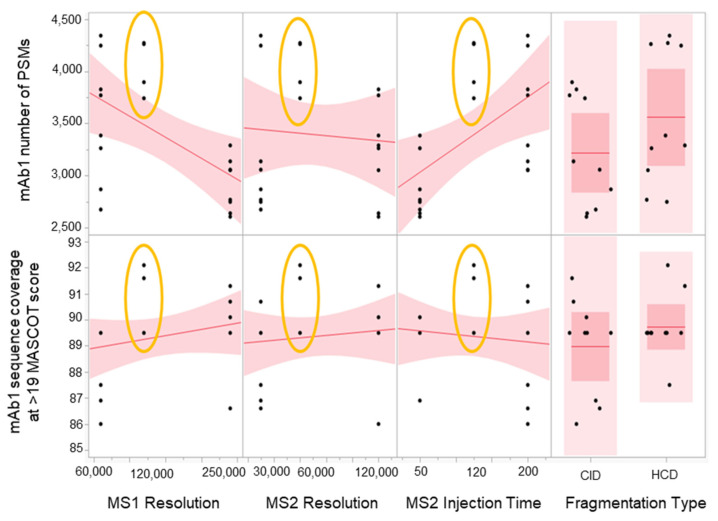
Scatterplot matrix showing the main effects of MS1 resolution, MS2 resolution, MS2 injection time, and fragmentation type on mAb1 PSMs and sequence coverage. Results from midpoints are encircled in yellow.

The results (Figure 4) show that the target conditions yielded the highest number of PSMs and the best sequence coverage for the low abundance mAb1. This suggests that the parameters chosen were optimal. In addition, there was less variability with the results from the target conditions compared to the higher and lower conditions, which suggests that the method is robust. There was also no significant advantage between using CID vs. HCD. We have chosen to go with CID for the final method since this fragmentation technique can produce more -b and -y ion series fragments compared to HCD, which was known to be -y ion biased [34,35]. A more comprehensive peptide fragment spectrum is ideal in SVA since it is important to have as complete of a fragment sequence coverage as possible for identification purposes. However, if CID was not available, HCD would have been appropriate as well.

### 2.4. Comparison of Relative Quantitation between Commonly Used Orbitrap Platforms

The Orbitrap Fusion Lumos MS is a high-end and expensive instrument that is usually only available in specialized laboratories. Other more common Orbitrap platforms such as the Q-Exactive and the Exploris series should be appropriate for SVA analysis as well since they are already commonly used for multi-attribute PTM workflows within the biopharma industry [36,37]. The 0.5% mAb1 spiked into mAb2 samples were analyzed with a similar high-resolution Top 5 DDA method using the Orbitrap Elite, Fusion Lumos, Q-Exactive, and Exploris 480 mass spectrometers. mAbs 1 and 2 are both of the IgG1 isotype and have a significant portion of shared sequences. Between the two mAbs, we determined six peptide pairs that have roughly the same peptide length and mass to act as surrogates for wildtypes (mAb2) and PTMs/SVs (mAb1). The comparison showed that the Top 5 DDA SVA method was able to obtain similar relative quantitation for each peptide pair; thus, any of these other platforms might be suitable for SVA analysis following some method optimization around relative quantitation (Figure 3). One difference to consider, however, will be the resolution and mass error differences between these platforms. Unlike Lumos and Exploris MS, the Orbitrap Elite and Q Exactive series do not have the Easy-IC capability to perform self-internal mass calibration for better mass accuracy. This will impact the minimum mass error tolerance one might pick during the MS/MS database search and subsequently the number of false positives generated during SV analysis. Additionally, the MS1 resolution used in the Elite, and Q-Exactive instruments had to be reduced to ensure that there will be enough scans across the chromatographic peak. Therefore, to minimize the number of false positives, an easy-IC equipped faster higher scan rate Orbitrap instrument is highly recommended.

### 2.5. PMI Byos SVA Parameter Selection

Twenty-three previously identified sequence variants from four SVA projects using the previously established Mascot workflow served as a benchmark to find suitable Byonic parameters for SVA. Like Mascot, Byonic uses a scoring system to determine PSM correctness. The score is a sum of “benefits” for theoretical peaks found in the (modified) observed spectrum and “penalties” for theoretical peaks that are not found [38]. Typical “good” PSMs will have a score of around 200–400 [39]. We tested Byonic peptide score cutoffs of 200, 250, and 300 and compared them with the Mascot cutoff score of 15, which we previously used to filter the data before further evaluation.

The optimized Byonic score should detect and positively identify one or more PSMs corresponding to each true sequence variant while minimizing the total number of unique peptide hits (true and false SVs) that will be subjected to manual evaluation. Among the three Byonic score cutoffs tested, the score of 250 provided the least total number of unique peptides with a sufficient number of PSMs. At the score of 300, the number of PSMs started to drop dramatically for mAb III and failed to detect one PSM corresponding to a sequence variant in mAb IV (Supplementary Table S2). Hence, 250 is the proposed Byonic score cutoff for SV identifications since this is the parameter that achieved equivalent sensitivity as that of the previously employed Mascot parameters and was able to decrease the number of unique peptides for manual inspection.

To further test the new score cutoff, mAb6 was spiked into mAb7 at 0.1, 0.2, and 0.5% (gram/gram) to mimic low-level sequence variants and was analyzed with an Orbitrap Fusion MS. This study was performed to evaluate the ability of Byonic to identify low levels of expected sequence variants (seven in total) and to confirm the proposed score cutoff of 250.

The results in Supplementary Table S3 show that, with a Byonic score of 225, all seven sequence variants were still detected at 0.2%. At the previously proposed cutoff of 250, only four out of seven sequence variants were detected at 0.2%. This was not acceptable since the SVA assay needs to be able to detect all sequence variants at levels ≥0.2%. Therefore, 225 was selected as the Byonic cutoff score for the new high-resolution sequence variant workflow.

### 2.6. Direct Comparison of Low-Resolution vs. High-Resolution Methods

To determine the performance of the newly developed method, a side-by-side comparison of the low- and high-resolution methods was performed using real SVA samples without spiked proteins.

Initially, the MS1 mass tolerance was set to a more conservative 5 ppm while the MS2 mass tolerance was set to 10 ppm for the high-resolution method. The high-resolution SVA method detected all of the previously identified sequence variants and maintained high sequence coverage while reducing the total number of unique peptides by 76–90% among the molecules tested (Table 2).

The high-resolution MS method resulted in fewer PSMs corresponding to the positively identified sequence variants (Table 3) but was still able to detect all expected sequence variants with more than one PSM each. Having more than one PSM corresponding to a proposed sequence variant provides confidence in the positive SV identification. There was also similar relative quantitation for each of the identified sequence variants between the low- and high-resolution SVA methods.

Since the high-resolution method used the Easy-IC internal calibration feature available on the Fusion Lumos MS, it was expected that the MS1 mass error should be <1 ppm for any given scan. This implies that there might be an opportunity to further constrict the mass tolerances employed for the SVA database search in PMI-Byos. We tested this by lowering the mass tolerance for MS1 to 2 ppm from 5 ppm and for MS2 from 10 ppm to 5 ppm. This should be well within the capability of the instrument if the manufacturer’s claim of <1 ppm mass accuracy per scan is true. Applying these tightened parameters cut (0 to ~50%, depending on the molecule) the number of unique peptides even further and did not compromise the identification of any true sequence variants. The number of unique peptides for mAbs 3, 4, and 5 went from 83, 43, and 30 to 86, 24, and 22, respectively (Supplementary Table S4).

The improved mass spectrometer technology and data analysis software allowed us to apply a combination of high resolution MS/MS and improved software to directly improve upon Yang et al.’s reported method [17]. With the greatly reduced SV candidate PSMs, we are now able to reduce the total SVA workflow from sample preparation to LC-MS data acquisition, data analysis, data review, electronic notebook signing off, and SVA Memo release from 6 to 8 weeks to just 2 weeks. This analysis speed is comparable to that of NGS [15]. While NGS can only detect SVs from genetic mutations, SVA by LC-MS/MS has the advantage of being able to detect both genetic and translation-derived sequence variants. While this takes longer than the recently reported new peak detection-based SVA method [40], this method works without the need for any pre-annotated reference standards for comparison and can be used de novo for all types of protein therapeutics.

## 3. Material and Methods

### 3.1. Material

This study used various therapeutic proteins produced at Genentech’s facility (South San Francisco, CA, USA). mAb1, mAb2, mAb4, mAb5, mAb6, mAb7, and mAb IV are recombinant humanized recombinant IgG1 monoclonal antibodies expressed in Chinese hamster ovary (CHO) cells. mAb3, mAb I, mAb II, and mAb III are recombinant humanized IgG1 Fab fragments expressed in E coli. Fusion protein 1 (FP1) is an Fc-fusion protein expressed in CHO cells. All therapeutic proteins were purified by standard manufacturing procedures. All organic solvents were LC-MS grade.

### 3.2. Methods

#### 3.2.1. Peptide Map by Low-Resolution MS/MS

The therapeutic proteins were analyzed by a combination of tryptic, Asp-N, and thermolysin peptide mapping. The protein was digested with trypsin (Roche recombinant proteomics grade, Indianapolis, IN, USA), Asp-N (Roche sequencing grade, Indianapolis, IN, USA), and thermolysin (Sigma, St. Louis, MO, USA) in separate tubes (protein: enzyme ratio of 50:1, 125:1, and 100:1, respectively) after being subjected to denaturation, reduction, and alkylation with heavy-labeled (C^13^) iodoacetic acid (Cambridge Isotope Laboratories, Tewksbury, MA, USA). The heavy-labeled isotope was used to distinguish alkylation derivatives from Ala->Glu and Gly->Asp substitutions, which have an isobaric mass shift (+58.0055 Da). The peptide mixture was separated and analyzed using an Acquity (H-Class) UHPLC (Waters Corporation, Milford, MA, USA) coupled with an Orbitrap Elite MS (Thermo Fisher Scientific, Sunnyvale, CA, USA). The protein digest (20 μg) was injected into an Acquity UPLC Peptide CSH C18 Column (2.1 mm I.D. × 150 mm, 1.7 µm, 130 Å) (Waters Corporation, Milford, MA, USA) and was used with a column temperature of 77 °C. Mobile phases A and B consisted of 0.1% LCMS-grade formic acid in water (JT Baker, Phillipsburg, NJ, USA) (A) and 0.1% LCMS-grade formic acid in acetonitrile (JT Baker, Phillipsburg, NJ, USA) (B). After holding at 0% B for 2 min, a linear gradient was run from 0% to 15% B in 10 min, to 32% in 73 min, and 50% in 13 min, followed by a wash step 95% B for 3 min before column re-equilibrium at the initial condition (20 min). The flow rate was set to 0.2 mL/min.

Tandem mass spectrometry. The separated peptides from the UPLC were eluted into the Orbitrap Elite ESI-MS operating in a positive ion mode with a mass scan range of mass-to-charge ratio (*m*/*z*) 200–2000. For the MS/MS product ion scan, the activation type was collision-induced dissociation (CID); the normalized collision energy was 35; and the activation time was 10 ms. The MS method consists of a full MS survey scan event in the Orbitrap at 60 K FWHM and an MS/MS turbo scan on the top five most intense ions in the Ion trap. The dynamic exclusion (DE) function was enabled to reduce data redundancy and allow low-intensity ions to be selected for data-dependent scans. The DE parameters were as follows: a repeat duration of 30 s, an exclusion list size of 500, an exclusion duration of 45 s, a low and high mass width of 10 ppm, and a repeat count of 4.

Mascot MS/MS search. The .raw data file obtained from the MS analysis was filtered to contain only the MS/MS scans and converted to .mgf file format using the msconvert program (ProteoWizard). Next, the .mgf file was uploaded to an on-premise Mascot server for error-tolerant search (ETS). The search parameters used were the following: max missed cleavages was 1; peptide tolerance was ±8 ppm; MS/MS tolerance was ±0.8 Da; data format was set as mascot generic; instrument was set to ESI-TRAP; and only Top 5 hits were reported. The Mascot ETS search results were then fed into PMI ISSA software for manual inspection and verification of proposed sequence variants.

An R script was written to automatically annotate these false positives in ISSA or Byologic, similar to the approach described by Li et al. [18]. The script first reads a sequence variant analysis ISSA or Byologic project file (SQLite) and then annotates each potential sequence variant assignment that meets at least one of the specified conditions as “false positive”. The script then provides a description of the conditions that led to each annotation in the comments field for the respective sequence variant assignment. All other potential sequence variant assignments are left unchanged. The conditions used to identify incorrectly assigned sequence variants are based on previously observed false positives. These conditions are the following: 1. Sequence variants from the trypsin digest that are on semi-tryptic peptides where Arg or Lys are not involved in the called sequence variant. 2. Sequence variants where the mass shift is equal to that of an amino acid, and that amino acid is present near one of the peptide termini. 3. Sequence variants that are below a pre-specified quantification limit. 4. Sequence variants from orthogonal digests that are already well covered by the trypsin digest.

#### 3.2.2. High Resolution MS Parameter Optimization and Robustness Testing

JMP version 14 (SAS Institute, Cary, NC, USA) was used for the DOE performed to select and test the robustness of the Orbitrap Fusion Lumos MS parameters. First, a four-factor, two-level fractional factorial screening design was applied to select ion intensity thresholds and AGC targets and to investigate main factors and possible two-way interactions. Three- or more-factor interactions are extremely rare in practice and were thus assumed to be nonexistent [41]. The maximum and minimum values for each parameter are summarized in Table 4.

Second, a four-factor, two-level fractional factorial design was selected to test the robustness of the proposed high-resolution MS method. In this study, the midpoint values are the target parameter conditions. The maximum MS/MS injection time was increased to 200 ms based on the results of the screening DOE study. The effect of CID and Higher-energy C-trap dissociation (HCD) MS/MS fragmentation techniques were also investigated in this study (Supplementary Table S1).

For both DOE studies, the sample was used as a tryptic digest of mAb1 spiked into mAb2 at 0.5% (gram/gram) and followed the digestion, chromatography, and MS/MS search conditions described previously. These two mAbs are both IgG1s and share a large percentage of sequences except in a few sites. The response factors used to evaluate the parameter screening DOE results are mAb1 and mAb2 sequence coverage at >19 Mascot score, total aggregate mAb1 and mAb2 scores, and the total number of MS/MS scans. The response factors used to evaluate the method robustness DOE are the number of peptide spectrum matches (PSMs) and the sequence coverage for mAb1 at >19 Mascot score.

#### 3.2.3. PMI Byos Parameter Selection

First, four previously analyzed SVA samples containing twenty-three total true positive sequence variants were identified using Mascot and served as a benchmark to fine-tune the Byonic search parameters and to optimize the score threshold in Byonic. To test the parameters chosen in the first study, a second study was conducted where mAb6 was spiked into a mAb with similar a structure (mAb7) at 0.1, 0.2, and 0.5% (gram/gram) to mimic true low-level sequence variants. mAbs6 and mAbs7 only differ in a few sites and will yield seven peptides with true positive sequence variants.

#### 3.2.4. Peptide Map by High-Resolution LC-MS/MS

This method uses the same sample preparation and UHPLC conditions as the low-resolution method.

Tandem mass spectrometry. The separated peptides from the UPLC were eluted into the Orbitrap Fusion Lumos ESI-MS operating in a positive ion mode with a mass scan range of mass-to-charge ratio (*m*/*z*) 200–2000. For the MS/MS product ion scan, the isolation mode was set to quadrupole; the monoisotopic precursor selection (MIPS) was set to peptide; the activation type was CID; the normalized collision energy was 35; the MS/MS injection time was 120 ms; the activation time was 10 ms; and the activation Q was 0.25. The MS method consists of a full MS survey scan event in the Orbitrap at 120 K resolution and 4 × 10^5^ AGC target and of an MS/MS scan on the top five most intense ions in the Orbitrap at 60 K resolution and 5 × 10^4^ AGC target. The dynamic exclusion (DE) function was enabled to reduce data redundancy and allow low-intensity ions to be selected for data-dependent scans. The DE parameters were as follows: a repeat duration of 30 s, an exclusion duration of 5 s, a low and high mass width of 10 ppm, and a repeat count of 1. The Easy-IC internal calibration feature was enabled in MS1 to achieve precursor mass accuracies <1 ppm [24].

PMI Byos Sequence Variant Analysis Workflow. A custom workflow with three separate parameters for each enzyme (trypsin, AspN, thermolysin) was created for the sequence variant database search. For general instrument parameters, the mass tolerance was set to 2.0 ppm, and the fragmentation type was set to CID; the fragmentation mass tolerance was set to 5.0 ppm. The digestion cleavage site parameter was set according to each enzyme’s specificity. For trypsin, a semi-specific search was selected. For thermolysin, the cleavage site was set to the N-terminal of amino acids A, F, I, L, M, and V with unlimited missed cleavages. The MS extraction window was set to 10 ppm.

The same R-script for MASCOT search results was used to perform automated filtering and false positive annotation for the resulting proposed sequence variant hits from PMI Byos SVA.

## 4. Conclusions

We have developed an improved high-resolution LC-MS/MS SVA method that took advantage of recent advances in MS technology and data analysis software and was able to eliminate up to 93% of false positives automatically, greatly increasing the SVA efficiency. In the process, we discovered that the IIT feature of the Fusion Lumos caused significant relative under-quantitation of low-level peptides and turned it off. A comparison of common Orbitrap platforms showed that similar quantitation values were obtained on a spiked-in sample. This newly developed SVA workflow has a short turnaround SVA time (~2 weeks) similar to NGS but can detect both SVs from either genetic mutations or translation errors, making LC-MS/MS analysis as the top choice for SVA.

## Figures and Tables

**Table 1:** Effect of MS/MS Injection Time on Scores and Coverage.

MS/MS Injection Time (ms)	mAb1 Aggregate Score	mAb1 Sequence Coverage	mAb2 Aggregate Score	mAb2 Sequence Coverage
100	1662	88%	14,924	90%
150	2222	89%	25,304	93%
200	2302	89%	31,381	93%
250	2557	89%	34,722	94%

**Table 2:** High vs. low-resolution SVA unique peptides, detected SVs, and sequence coverage comparison.

Molecule Name		High Resolution	Low Resolution	% Reduction
mAb3	Unique Peptides	83	346	76%
	Number of true SVs	5	5	
	Sequence Coverage	100	100	
mAb4	Unique Peptides	43	194	78%
	Number of true SVs	1	1	
	Sequence Coverage	100	100	
mAb5	Unique Peptides	30	298	90%
	Number of true SVs	0	0	
	Sequence Coverage	100	99	
FP1 ^1^	Unique Peptides	49	458	89%
	Number of true SVs	15	15	
	Sequence Coverage	91 ^2^	92 ^2^	

^1^ Tryptic map only, ^2^ Reduced sequence coverage due to missing small hydrophilic peptides generated by trypsin.

**Table 3:** High vs. low-resolution SVA method quantitation and PSM comparison.

Molecule	Sequence Variant	High Resolution		Low Resolution	
		Rel. Quant (%)	Number of PSMs	Rel. Quant (%)	Number of PSMs
mAb3	D1E_LC	0.3	9	0.2	18
	D17E_LC	0.3	10	0.3	3
	G128E_LC	0.3	6	0.2	18
	I48Mnl ^1^_LC	0.2	4	0.2	8
	I70Mnl ^1^_HC	0.2	7	0.2	12
mAb4	T20I_LC	0.9	3	0.9	9
mAb5	N/A ^2^	N/A	N/A	N/A	N/A
FP1 ^3^	Y58F	0.7	18	0.8	20
	Y76F	0.7	2	0.8	2
	Y80F	1	2	1.2	2
	Y129F	0.8	32	0.8	16
	Y153F	1	20	1.5	10
	Y171F	1	18	1.5	10
	Y187F	0.9	8	1.2	10
	Y216F	0.6	8	0.7	12
	Y276F	5	4	8.8	8
	Y280F	0.7	12	0.8	16
	Y312F	0.3	20	0.2	112
	Y317F	0.9	18	1.1	152
	Y326F	0.7	20	0.9	23
	Y329F	0.8	12	0.8	9
	Y336F	0.5	10	0.6	10

^1^ Mnl is methylnorleucine, ^2^ Not applicable because mAb5 has no sequence variants, ^3^ FP1 has heavy Tyr->Phe misincorporation.

**Table 4:** Maximum, minimum, and midpoint values used for each of the MS parameters in the screening DOE study.

Parameter	Units	Minimum (−)	Maximum (+)	Midpoint (0)
DDA Intensity Threshold	ions	50,000	500,000	275,000
MS/MS Injection Time	ms	100	35	67.5
MS/MS AGC Target	ions	100,000	500,000	300,000
MS1 AGC Target	ions	1,000,000	5,000,000	3,000,000

## Data Availability

Not applicable.

## Supplementary Materials

The following supporting information can be downloaded at: https://www.mdpi.com/article/10.3390/molecules28083392/s1, Figure S1: Pareto plot of estimates showing the relative magnitude of the effects of DDA threshold, MS2 injection time, MS1 AGC target and MS2 AGC targets; Figure S2: Comparison of 0.1% vs. 0.5% mAb1 spiked-in mAb 2: (A) The sequence coverage of both samples were similar at around ~92%; (B) all six quantitative pairs were detected at levels close to the theoretical relative quantitation of 0.1%; Table S1: Maximum, minimum, and midpoint values used for each of the MS parameters in the robustness DOE study; Table S2: Search Engine Comparison-Mascot vs. PMI Byonic; Table S3: Number of detected sequence variants, unique peptides, and PSMs by Mascot vs. Byonic; Table S4: SV hits obtained used different precursor mass error tolerances.
