# Supplementary material for: A Highly Efficient Workflow for Detecting and Identifying Sequence Variants in Therapeutic Proteins with a High Resolution LC-MS/MS Method
Source: Molecules. 2023 Apr 12;28(8):3392. doi: 10.3390/molecules28083392 (PMC10144261; doi:10.3390/molecules28083392)
Supplement: Supplementary file 1 [file molecules-28-03392-s001.zip › molecules-2316356-supplementary.pdf]

SUPPLEMENTARY MATERIAL

A Highly Efficient Workflow for Detecting and Identifying Sequence Variants in Therapeutic Proteins with a High Resolution LC-MS/MS Method

Lance Cadang [1], Chi Yan Janet Tam [1], Benjamin Nathan Moore [1], Juergen Fichtl [2] and Feng Yang [1,*]

[1] Pharma Technical Development, Genentech, South San Francisco, CA 94080 USA
[2] Pharma Technical Development, Roche Diagnostics GmbH, 82377 Penzberg, Germany
* Correspondence: yang.feng@gene.com

Supplementary Table S1. Maximum, minimum, and midpoint values used for each of the MS parameters in the robustness DOE study

Parameter	Units	Minimum (-)	Maximum (+)	Midpoint (0)
MS1 Resolution	FWHM @ m/z 200	60000	240000	120000
MS/MS Resolution	FWHM @ m/z 200	30000	120000	60000
MS/MS Injection Time	ms	50	200	125
MS Activation	N/A	CID	HCD	N/A

Supplementary Table S2. Search Engine Comparison - Mascot vs. PMI Byonic

SVA Project	Number of SVs	Mascot Score @15		Byonic Score @200		Byonic Score @250		Byonic Score @300	
		#Unique Peptides	#SV PSMs	#Unique Peptides	#SV PSMs	#Unique Peptides	#SV PSMs	#Unique Peptides	#SV PSMs
mAb I	5	623	37	643	26	525	23	428	22
mAb II	11	317	46	405	46	326	44	267	42
mAb III	6	145	23	322	24	232	23	177	14
mAb IV	1	241	1	237	1	184	1	142	0

Supplementary Table S3. Number of detected sequence variants, unique peptides, and PSMs by Mascot vs. Byonic

Spike %	Mascot Score @15			Byonic Score @200			Byonic Score @225			Byonic Score @250			Byonic Score @300		
	#SVs	#Unique Peptide	#PSMs	#SVs	#Unique Peptide	#PSMs	#SVs	#Unique Peptide	#PSMs	#SVs	#Unique Peptide	#PSMs	#SVs	#Unique Peptide	#PSMs
0.5	6	3775	33	7	1411	32	7	1239	30	7	1068	30	7	793	26
0.2	5	4082	14	7	1462	20	7	1261	18	4	1076	13	4	807	12
0.1	5	3122	14	5	1380	17	3	1186	14	3	1020	14	2	743	7

Supplementary Table S4. SV hits obtained per SVA of mAbs 3, 4, and 5 using either a 10 ppm MS1 5ppm MS2 or 2 ppm MS1 5 ppm MS2 mass error tolerance in the Byos MS/MS search.

Sample	Lumos (10, 5)	Lumos (2, 5)	% Further Reduction
mAb3	83	86	-4%
mAb4	43	20	53%
mAb5	30	22	27%

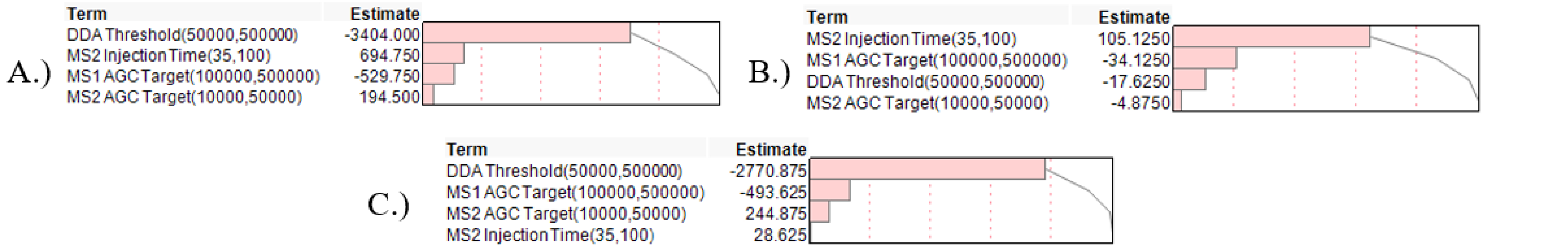

Supplementary Figure S1. Pareto plot of estimates showing the relative magnitude of the effects of DDA threshold, MS2 injection time, MS1 AGC target and MS2 AGC target to the abundant peptide aggregate (A) score, low-level peptide aggregate score (B), and total MS/MS queries (C).

A.)

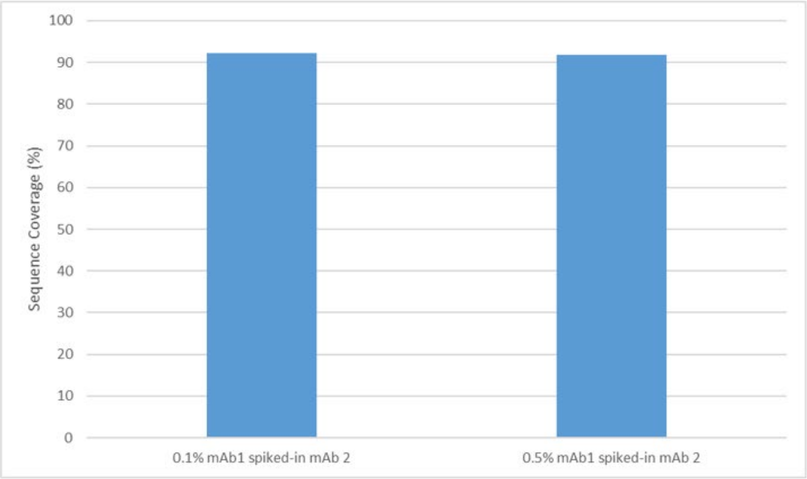

B.)

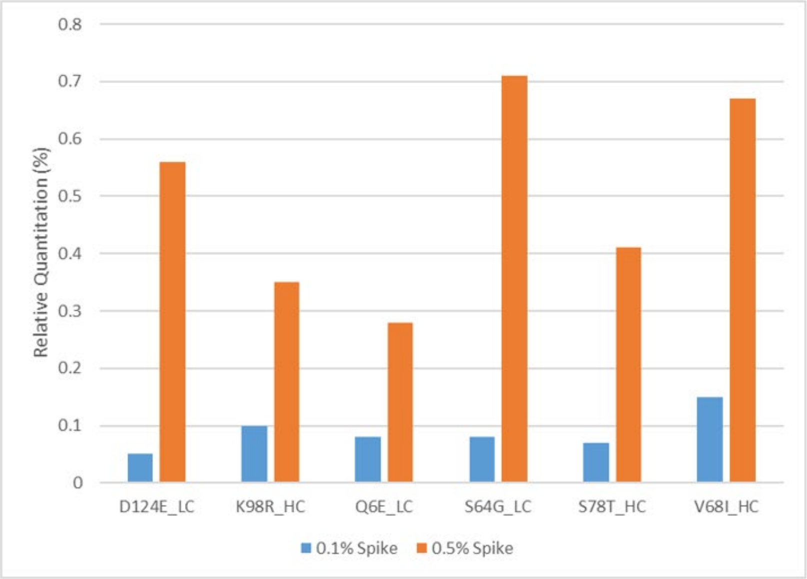

Supplementary Figure S2. Comparison of 0.1% vs 0.5% mAb1 spiked-in mAb 2: (A) The sequence coverage of both samples were similar at around ~92%; (B) all six quantitative pairs were detected at levels close to the theoretical relative quantitation of 0.1%.
